# Is aggressive care appropriate for patients with cancer complicated by pneumonia? A retrospective chart review in a tertiary hospital

**DOI:** 10.1186/s12904-023-01127-2

**Published:** 2023-01-06

**Authors:** Chanchanok Aramrat, Thawalrat Ratanasiri, Patama Gomutbutra

**Affiliations:** 1 Department of Family Medicine, Faculty of Medicine, Chiang Mai University, 110 Intawaroros Road, Tambon Sriphum, Muang District, Chiang Mai, 50200 Thailand; 2 Karunruk Palliative Care Center, Faculty of Medicine, Srinagarind Hospital, Khon Kaen University, 123 Mittraphap Road, Tambon Nai-Muang, Muang District, Khon Kaen, 40002 Thailand

**Keywords:** Cancer, Pneumonia, In-hospital mortality rate, Prognosis, Lymphocyte, Oxygen pulse

## Abstract

**Background:**

Pneumonia in cancer patients is often problematic in order to decide whether to admit and administer antibiotics or pursue a comfort care pathway that may avoid in-hospital death. We aimed to identify factors which are easily assessed at admission in Thailand’s healthcare context that could serve as prognostic factors for in-hospital death.

**Methods:**

Regression analysis was utilized to identify the prognostic factors from clinical factors collected at admission. The primary outcome was in-hospital death. Data was collected from the electronic medical records of Chiang Mai University Hospital, Thailand, from 2016 to 2017. Data on adult cancer patients admitted due to pneumonia were reviewed.

**Results:**

In total, 245 patients were included, and 146 (59.6%) were male. The median age of the patients was 66 years (IQR: 57–75). A total of 72 (29.4%) patients died during admission. From multivariate logistic regression, prognostic factors for in-hospital death included: Palliative Performance Scale (PPS) ≤ 30 (OR: 8.47, 95% CI: 3.47–20.66), Palliative Performance Scale 40–50% (OR: 2.79, 95% CI: 1.34–5.81), percentage of lymphocytes ≤ 8.0% (OR: 2.10, 95% CI: 1.08–4.08), and pulse oximetry ≤ 90% (OR: 2.01, 95% CI: 1.04–3.87).

**Conclusion:**

The in-hospital death rate of cancer patients admitted with pneumonia was approximately 30%. The PPS of 10–30%, PPS of 40–50%, percentage of lymphocytes ≤ 8%, and oxygen saturation < 90% could serve as prognostic factors for in-hospital death. Further prospective studies are needed to investigate the usefulness of these factors.

**Supplementary Information:**

The online version contains supplementary material available at 10.1186/s12904-023-01127-2.

## Key Statements

### What is already known about the topic?

- Scoring systems developed specifically for cancer patients require information that may not be available in all Thailand’s hospital settings.
- CURB-65 and Pneumonia Severity Index (PSI) for predicting mortality in pneumonia patients usually are not accurate predictors for cancer patients.

### What this paper adds

- This study found that the Palliative Performance Scale (PPS) ≤ 30%, PPS 40–50%, percentage of lymphocytes ≤ 8.0%, and pulse oximetry ≤ 90% were found to be potential prognostic factors for in-hospital death in this group of patients.

### Implications for practice, theory, or policy

- This study suggests prognostic factors for guiding management in cancer patients with acute pneumonia, however, prospective studies are needed to confirm this hypothesis.

## Background

The prognosis of inevitable death in cancer patients, despite aggressive treatment, is important for decision-making as 70% of Thai people prefer to die at home [1]. The decision not to pursue further aggressive treatment may allow patients to spend their last moments in place where they feel most comfortable.

Pneumonia is one of the most common causes of hospitalization in patients with cancer, and its prognosis differs from that of the general population [2]. In the general population, scoring systems have been developed to predict mortality in patients with pneumonia. Two well-known scoring systems are Pneumonia Severity Index (PSI) [3] and the CURB-65 [4]. The PSI score consists of 20 clinical parameters that may be cumbersome for use in clinical practice, especially in Thailand’s hospital settings. The CURB-65 requires only five clinical parameters, however these scoring systems have not performed well in the cancer population [5, 6]. Some studies have explored other prognostic factors in cancer patients. For example, Ahn et al. [6] found lactic acid levels better at predicting 28-day mortality compared to PSI or CURB-65. However, some of these factors are not routinely assessed in Thai hospitals. For example, lactic acid levels are not usually assessed in pneumonia patients with relatively stable vital signs at admission.

Therefore, our main objective is to identify potential prognostic factors for in-hospital death that are usually assessed at the time of admission in Thailand’s hospital settings. We chose in-hospital death as our outcome because the place of death is a major concern for end-of-life palliative patients. The findings from this study may apply to other low to middle-income countries’ hospital settings as well.

## Methods

### Study design and population

This was a retrospective cohort study that reviewed data from Chiang Mai University Hospital’s electronic database on adult cancer patients admitted between January 1, 2016, and December 31, 2017. Chiang Mai University Hospital is a 1,400-beds hospital, serves as a tertiary referral center in northern Thailand.

Admissions of patients who were diagnosed with cancer and pneumonia and those greater than 14 years of age were electronically extracted from the electronic medical records. Cancer patient admissions were determined with one of the following the ICD 10 [7]  diagnoses: C00-C96. Pneumonia patient admissions were determined with one of the following ICD 10 diagnoses: J12-J18, J69, and J82. All extracted admissions were manually reviewed by authors (C.A. and T.R.). A patient was considered to have cancer with a histological or cytological confirmation of malignancy before admission. In addition, patients who received cancer treatment before admission, or the latest radiologic image before admission showed evidence of cancerous tissue were also included. A patient was considered to have pneumonia at the time of admission if at least one of the following clinical symptoms was present at the time: cough, fever, sputum production, or pleuritic chest pain, with new lung infiltration on radiological imaging [8].

Admissions that developed pneumonia during admission were excluded. Admissions treated elsewhere for pneumonia before referral to Chiang Mai University Hospital were also excluded. With multiple admissions of the same individual, only the first admission was included. Patients with documentation of advanced care directives before or at the time of admission were also excluded. This was done to ensure that the entry data was relevant to the research question and to minimize the effect of any potential confounders.

During the review process, any disagreement between the authors was settled by discussion until both reached the same conclusion. Unresolved issues were determined by the third author (PG).

### Data collection

Factors associated with mortality rates in patients with pneumonia and/or cancer have been reviewed [3, 4, 6, 9–12]. The factors which can be easily assessed in Thailand’s hospital settings including age, gender, type and stage of cancer, comorbidities (congestive heart failure, cerebrovascular disease, renal disease, and liver disease), vital signs (pulse rate, respiratory rate, blood pressure, body temperature, and oxygen saturation), and state of confusion. Additional data includes the Palliative Performance Scale (PPS), a tool for measuring the performance status of palliative care patients [13], within the first 24 h of admission using the Thai translated version [14]; complete blood count; serum sodium; serum glucose and blood urea nitrogen. The history of non-surgical cancer treatment, including chemotherapy, and/or radiotherapy in chest area within four weeks before admission were also collected. Blood lactic acid levels and arterial pH were not included in this study as they are not accessible in every hospital in Thailand. Status at discharge and length of stay were collected. The electronic records were manually reviewed. All extracted data were anonymized and de-identified before analysis.

### Statistical analysis

**Fig. 1:**
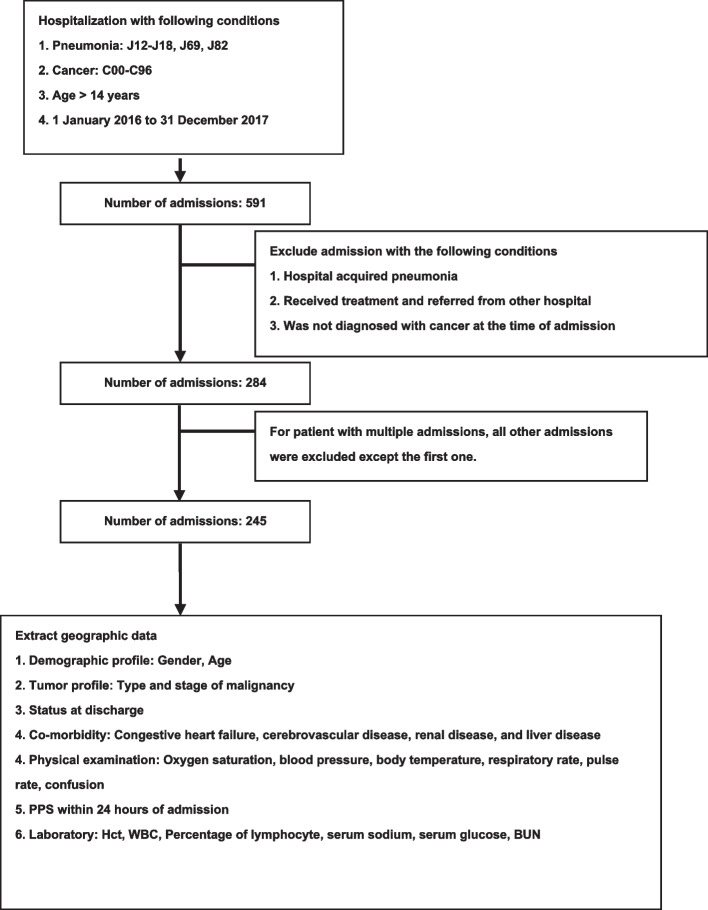
Inclusion and exclusion flow diagram

Patient demographics were descriptively analyzed. Continuous variables were expressed as medians and interquartile ranges (IQR). Categorical variables were expressed as absolute numbers and percentage frequencies. The proportion of in-hospital deaths was calculated. All factors were categorized into categorical variables following previously published studies related to pneumonia mortality [3, 4, 6, 9] before proceeding to univariate logistic regression analysis. PPS were grouped following Clement Ma et al. study [15]. The cutoff point for the percentage of lymphocytes was less than or equal to 8.0%, as this was the median value for the factor in our study. Only factors with a *p*-value < 0.10 were candidates for multivariate logistic regression analysis. Factors with a *p*-value > 0.05 were systematically eliminated by the backward stepwise method. A complete case analysis was conducted in this study and missing data were not imputed. Factors with missing data of more than 20% were excluded from the analysis. Model goodness-of-fit was tested with the Hosmer-Lemeshow test. A two-tailed p-value of less than 0.05 was considered statistically significant. Stata version 15.1 was used for the statistical analysis. The overview of the study methodology is shown in Fig. 1.

## Results

### Baseline characteristics

A total of 245 patients were included in the analysis with a median patient age of 66 years (IQR 57–75), of which 146 were male patients (59.6%). Of these cases, 195 (79.6%) patients had solid tumor, 48 (19.6%) had hematologic malignancy and 2 (0.8%) had metastatic cancer without tissue confirmation of the origin of cancer. Of all the included patients, only 184 had documentation of cancer staging during their admission. There are 4 patients in Stages 1, 4 patients in stage 2, 18 patients in Stage 3, 142 patients in Stage 4, 4 patients were documented as advanced cancer, and 12 were documented as recurrent/relapse of cancer. The four most diagnosed cancers were malignant neoplasms of the respiratory and intrathoracic organs (96 patients, 39.2%); malignant neoplasms of lymphoid, hematopoietic, and related tissue (48 patients, 19.6%); malignant neoplasms of the digestive organs (35 patients, 14.3%); and malignant neoplasms of the lip, oral cavity, and/or pharynx (14 patients, 5.7%).

### In-hospital death rate

Of the 245 patients, 173 were alive at the time of discharge, and 72 died during the hospital stay. The in-hospital mortality rate was calculated to be 29.4% (95% CI: 24.0-35.4%). The median lengths of stay for patients who were alive at the time of discharge was nine days (IQR: 6–14), those who died in hospital had a median stay of eight days (IQR: 4-16.5). The general characteristics of the patients are shown in Table 1.

**Table 1:** General population characteristics

	Median / frequency
Age in years [IQR]	66 [57–75]
Male (%)	146 (59.6)
**Type of Cancer**	
Solid tumor (%)	195 (79.6)
Hematologic malignancy (%)	48 (19.6)
Unknown origin (%)	2 (0.8)
**Vital signs**	
Pulse rate per minute [IQR]	112 [94–128]
Respiratory rate per minute [IQR]	24 [20–30]
Pulse oximetry in % [IQR]	90.5 [85.0–95.0]
Systolic BP in mmHg [IQR]	115 [101–134]
Diastolic BP in mmHg [IQR]	70 [61–81]
Body temperature in Celsius [IQR]	37.2 [36.6–38.2]
**PPS within 24 h of admission**	
PPS 100 (%)	0 (0.0)
PPS 80–90 (%)	15 (6.4)
PPS 60–70 (%)	103 (43.8)
PPS 40–50 (%)	78 (33.2)
PPS ≤ 30 (%)	39 (16.6)
**Laboratory**	
Hematocrit in % [IQR]	30.9 [27.0-35.8]
WBC per mm ^3^ [IQR]	11,600 [7,800 − 17,800]
Percentage of lymphocytes [IQR]	8.0 [5.1–14.6]
Serum sodium in mEq/L [IQR]	133 [129–136]
Serum glucose in mg/dL [IQR]	121 [102.5-157.5]
BUN in mg/dL [IQR]	17.0 [11.0–26.0]
**Comorbidities**	
Congestive heart failure (%)	2 (0.8)
Cerebrovascular disease (%)	7 (2.9)
Renal disease (%)	16 (6.5)
Liver disease (%)	8 (3.3)
**Treatment history**	
Received chemotherapy within 4 weeks (%)	50 (20.4)
Received chest radiation therapy within 4 weeks (%)	1 (0.4)

### Factors associated with mortality rate during admission

For the univariate logistic regression analysis, we combined patients with PPS 60–70, 80–90, and 100 together. Seven factors were identified to have p-values below 0.10, as follows: pulse oximetry ≤ 90% (OR: 2.37, 95% CI: 1.32–4.28), respiratory rate ≥ 30 per minute (OR: 2.70, 95% CI: 1.50–4.86), PPS 40–50 (OR: 3.52, 95% CI: 1.77-7.00), PPS 10–30 (OR: 9.51, 95% CI: 4.17–21.69), percentage of lymphocytes ≤ 8.0% (OR: 2.34, 95% CI: 1.33–4.15), BUN ≥ 20 mg/dL (OR: 2.22, 95% CI: 1.14–4.32), and history of chemotherapy within four weeks (OR: 0.39, 95% CI: 0.17–0.88). The results are shown in Table 2.

**Table 2:** Univariate logistic regression analysis

Variables	Univariate analysis		
	Odds ratio	95% CI	*p*-value
Patient demographic			
Age ≥ 65 years	1.01	0.99–1.03	0.319
Male	0.93	0.53–1.62	0.796
**Malignancy profile**			
Solid tumor	1.04	0.52–2.08	0.911
**Vital signs**			
Pulse oximetry ≤ 90%	2.37	1.32–4.28	0.004
RR ≥ 30 per minute	2.70	1.50–4.86	0.001
SBP < 90 mmHg or DBP ≤ 60 mmHg	0.78	0.41–1.48	0.449
PR ≥ 125 per minute	1.19	0.66–2.16	0.571
BT < 35 or ≥ 40 Celsius	0.59	0.07–5.40	0.643
**PPS within 24 h of admission** **(Compared to PPS ≥ 60)**			
PPS 40–50	3.52	1.77-7.00	< 0.001
PPS ≤ 30	9.51	4.17–21.69	< 0.001
**Laboratory**			
Hematocrit > 30%	0.91	0.52–1.59	0.745
WBC < 4 × 10^3 or > 20 × 10^3 per cubic mm	1.40	0.80–2.46	0.241
Percentage of lymphocytes ≤ 8.0%	2.34	1.33–4.15	0.003
Sodium < 130 mEq/L	1.16	0.63–2.12	0.636
Serum glucose ≥ 250 mg/dL	1.34	0.71–2.52	0.369
BUN ≥ 20 mg/dL	2.22	1.14–4.32	0.020
**Co-morbidity**			
Congestive heart failure^a^	-	-	-
Cerebrovascular disease	0.96	0.18–5.07	0.962
Renal disease	0.54	0.15–1.94	0.341
Liver disease	0.80	0.16–4.04	0.782
**Treatment history**			
Received chemotherapy within 4 weeks	0.39	0.17–0.88	0.023
Received chest radiation therapy within 4 weeks	-	-	-

^a^Both patients with congestive heart failure died during the hospital stay

All seven factors were entered into the multivariate logistic regression analysis. Factors were eliminated using backward elimination techniques at a *p*-value of > 0.05. The following four factors were found to be associated with in-hospital death rates: PPS 40–50 (OR: 2.79, 95% CI: 1.34–5.81), PPS ≤ 30 (OR: 8.47, 95% CI: 3.47–20.66), percentage of lymphocytes ≤ 8.0% (OR: 2.10, 95% CI: 1.08–4.08), and pulse oximetry ≤ 90% (OR: 2.01, 95% CI: 1.04–3.87) as seen in Table 3. The Hosmer-Lemeshow test indicated a well-fitted model (*p*-value 0.841).

**Table 3:** Multivariate logistic regression analysis

Variables	Multivariate analysis (*N* = 217)		
	Odds ratio	95% CI	*p*-value
**Vital signs**			
Pulse oximetry ≤ 90%	2.01	1.04–3.87	0.038
**PPS within 24 h of admission**			
PPS 40–50	2.79	1.34–5.81	0.006
PPS ≤ 30	8.47	3.47–20.66	< 0.001
**Laboratory**			
Percentage of lymphocytes ≤ 8.0%	2.10	1.08–4.08	0.029

## Discussion

In this study, the in-hospital death rate of cancer patients who were admitted with pneumonia between January 1, 2016, and December 31, 2017, was 29.4%. This rate is considered clinically high.

Similar studies by Gonzalez et al. [5] and Ahn et al. [6] found the 30- and 28-day mortality rates were 20.2% and 19.3%, respectively. Although the definition of mortality rate in this study was different from Gonzalez et al. and Ahn et al.‘s studies, they are roughly comparable. In this study more than 94% of the population were discharged within 28 days of admission, and our mortality rate did not account for patients who died after discharge. If we compare mortality rates using 28-day mortality rate definition, 29.39% would be an underestimation. A possible explanation for the large difference between studies is that ours only included admitted patients, whereas the other studies included outpatient cases as well. In addition, most of the patients included in our study had advanced cancer.

The four factors associated with the in-hospital death rate for cancer patients admitted with pneumonia were: PPS at 40–50%, PPS at 10–30%, percentage of lymphocytes ≤ 8.0%, and oxygen saturation ≤ 90%. Patient confusion was a factor that could not be assessed due to incomplete information in our electronic database.

The association between patient’s performance status and in-hospital mortality in our study was similar to Ahn et al.‘s study, [6] which also found that poor performance status was associated with the mortality rate. Although the Eastern Cooperative Oncology Group (ECOG) scale [16] was used to assess performance status in Ahn et al.‘s study, the ECOG and PPS can be used interchangeably [15, 17] PPS is a tool that is useful in estimating survival time in cancer patients [12]. The direction of association between PPS and the in-hospital mortality found in this study is also similar to the association between PPS and survival time observed in cancer patients.

The association between oxygen saturation and in-hospital mortality found in our study was also similar to Ahn et al.‘s [6] study. Oxygen saturation represents the ability of the lungs to oxygenate the blood. Low oxygen saturation is indicative of impaired lung function [18]. In this study population, impaired oxygen saturation would indicate severe pneumonia with a greater likelihood of death during the hospital stay.

Lymphocyte was found to be an important factor associated with the in-hospital mortality in this study. This finding is similar to Zhao et al. report [11]. Lymphocyte is a type of white blood cell responsible for reinforcing the immune system to fight infections [19, 20]. Moreover, lymphocytes are among the primary white blood cells to inhibit and kill cancer cells [20, 21]. Hence, they are important to the survivability of patients in this study. A decreased number of lymphocytes in cancer patients can be due to malnutrition, [22, 23] possibly due to the presence of cancer itself. Some cancer treatments can also decrease the number of lymphocytes, [24, 25] such as, radiation therapy and chemotherapy, making the body susceptible to numerous infectious attacks.

This study did not find a statistically significant association between the age of more than 65 years and the in-hospital mortality. However, studies have found that an age of more than 65 years was associated with mortality in the general population with pneumonia [3, 4]. Cancer treatments and cancer itself can weaken the immune system and overall body function [24, 25]. This process might be more pronounced than the effects of aging in the population with cancer, which may explain why age was not found to be associated with the in-hospital mortality in this study.

BUN level was found to be associated with the in-hospital mortality in the univariate logistic regression analysis but not in multivariate analysis. Ugajin et al. [26] proposed that the elevation of BUN levels in patients with pneumonia is probably due to dehydration. Under such conditions, the kidneys reabsorb urea along with water, which ultimately causes an elevation in BUN levels. However, in cancer population, performance status, lymphocytes, and oxygen saturation may be factors that are more closely associated to the mortality that attenuate association of BUN level.

## Limitations

This was a retrospective observational study done in a tertiary hospital in Chiang Mai as this study included large proportion of patients with advanced cancer it may skew the findings. This may limit the generalizability of our findings. Further studies are needed for external validation.

We used in-hospital death as our primary outcome while other studies focused on an overall 28-day mortality rate. In addition, consciousness and cancer staging were not included in our analysis model due to the high proportion of missing data. We also did not include chest x-rays findings in our study. Caution should be made when comparing this study’s results with others.

## Conclusion

In this study, the in-hospital death rate of cancer patients admitted with pneumonia was approximately 30%. The factors associated with the in-hospital death rate were PPS 10–30% and 40–50%, percentage of lymphocytes ≤ 8%, and oxygen saturation < 90%. These factors can easily be assessed in the Thailand’s hospital setting. The results of this study may be helpful in prognosis discussions for advanced cancer patients admitted with pneumonia.

## Supplementary Information

**Additional file 1.**

## Data Availability

The datasets generated during and analyzed during the current study are available from the corresponding author upon reasonable request.
