# Supplementary material for: Is aggressive care appropriate for patients with cancer complicated by pneumonia? A retrospective chart review in a tertiary hospital
Source: BMC Palliat Care. 2023 Jan 6;22:3. doi: 10.1186/s12904-023-01127-2 (PMC9817238; doi:10.1186/s12904-023-01127-2)
Supplement: Supplementary file 1 — Additional file 1. [file 12904_2023_1127_MOESM1_ESM.docx]

**Supplement material**

Binary transformation of variables, following other research papers related to pneumonia mortality

| **Variables** | **Threshold cut point** | **Reference** |
| --- | --- | --- |
| Age | ≥ 65 years | 1 |
| Pulse oximetry | ≤ 90 % | 2 |
| Respiratory rate | ≥ 30 per minute | 1 |
| Blood pressure | SBP < 90 mmHg  or DBP ≤ 60 mm Hg | 1 |
| Pulse rate | ≥ 125 beats per minute | 3 |
| Body temperature | < 35 or ≥ 40 Celsius | 3 |
| Hematocrit | < 30% | 3 |
| WBC | < 4x10^3^ per mm^3^  or > 20x10^3^ per mm^3^ | 4 |
| Sodium | < 130 mEq/L | 3 |
| Glucose | ≥ 250 mg/dL | 3 |
| BUN | ≥ 20 mg/dL | 1 |

1. Lim WS. Defining community acquired pneumonia severity on presentation to hospital: an international derivation and validation study. Thorax. 2003 May 1;58(5):377–82.

2. Ahn BK, Lee Y-S, Kim Y-J, et al. Prediction model for mortality in cancer patients with pneumonia: comparison with CURB-65 and PSI. Clin Respir J. 2018 Feb;12(2):538–46.

3. Fine MJ, Auble TE, Yealy DM, et al. A Prediction Rule to Identify Low-Risk Patients with Community-Acquired Pneumonia. N Engl J Med. 1997 Jan 23;336(4):243–50.

4. Community-acquired pneumonia in adults in British hospitals in 1982-1983: a survey of aetiology, mortality, prognostic factors, and outcome. The British Thoracic Society and the Public Health Laboratory Service. Q J Med. 1987;62(239):195-220.
